# Involvement of Akt-1 and mTOR in Sensitivity of Breast Cancer to Targeted Therapy

**DOI:** 10.18632/oncotarget.302

**Published:** 2011-07-01

**Authors:** Melissa L. Sokolosky, Kristin M. Stadelman, William H. Chappell, Stephen L. Abrams, Alberto M. Martelli, Franca Stivala, Massimo Libra, Ferdinando Nicoletti, Lyudmyla B. Drobot, Richard A. Franklin, Linda S. Steelman, James A. McCubrey

**Affiliations:** ^1^Department of Microbiology & Immunology, Brody School of Medicine at East Carolina University, Greenville, NC 27858 USA; ^2^These two authors contributed equally to the studies.; ^3^Dipartimento di Scienze Anatomiche Umane e Fisiopatologia dell'Apparato Locomotore, Università di Bologna, Bologna, Italy; ^4^IGM-CNR, Sezione di Bologna, C/o IOR, Bologna, Italy; ^5^Department of Biomedical Sciences, University of Catania, Catania, Italy; ^6^Palladin Institute of Biochemistry, National Academy of Sciences of Ukraine, Kyiv, Ukraine

**Keywords:** Akt, mTOR, Targeted Therapy, Drug Resistance

## Abstract

Elucidating the response of breast cancer cells to chemotherapeutic and hormonal based drugs is clearly important as these are frequently used therapeutic approaches. A signaling pathway often involved in chemo- and hormonal-resistance is the Ras/PI3K/PTEN/Akt/mTOR cascade. In the studies presented in this report, we have examined the effects of constitutive activation of Akt on the sensitivity of MCF-7 breast cancer cells to chemotherapeutic- and hormonal-based drugs as well as mTOR inhibitors. MCF-7 cells which expressed a constitutively-activated Akt-1 gene [ΔAkt-1(CA)] were more resistant to doxorubicin, etoposide and 4-OH-tamoxifen (4HT) than cells lacking ΔAkt-1(CA). Cells which expressed ΔAkt-1(CA) were hypersensitive to the mTOR inhibitor rapamycin. Furthermore, rapamycin lowered the IC_50_s for doxorubicin, etoposide and 4HT in the cells which expressed ΔAkt-1(CA), demonstrating a potential improved method for treating certain breast cancers which have deregulated PI3K/PTEN/Akt/mTOR signaling. Understanding how breast cancers respond to chemo- and hormonal-based therapies and the mechanisms by which they can become drug resistant may enhance our ability to treat breast cancer. These results also document the potential importance of knowledge of the mutations present in certain cancers which may permit more effective therapies.

## INTRODUCTION

Growth factor receptors are critical for controlling the proliferation of cells. Once activated, these receptors induce multiple signaling pathways involved in growth, prevention of apoptosis, differentiation and other fundamental processes. The epidermal growth factor (EGF) receptor (EGFR) and isoforms such as HER2 are also associated with breast cancer development and resistance to anticancer agents [1]. The HER2 growth factor receptor is frequently amplified in human breast cancer and is a target of diverse therapeutic approaches as well as an important biomarker [2-4]. Among the signaling pathways downstream of these and other receptors, the PI3K/PTEN/Akt/mTOR and Ras/Raf/MEK/ERK pathways have been shown to regulate growth and prevent apoptosis and their deregulation is often implicated in malignant transformation [5-12]. These pathways also play key roles in the genesis of cancer initiating cells (CICs, a.k.a. cancer stem cells) [13-22].

The PI3K/PTENAkt/mTOR pathway is frequently activated by mutations which may alter sensitivity to targeted therapy. The PI3K p110 catalytic subunit gene (*PIK3CA*) is one of the most frequently mutated genes in breast cancer [23]. Mutations in the kinase domain of *PIK3CA* will result in activation of Akt and contribute to malignant transformation, however the roles of these mutations in sensitivity to traditional chemo- and hormonal- based therapies and targeted therapies are not so well elucidated [24-26].

The regulation of Akt activation is complex. Akt is a key domino in determining whether a cell will survive and proliferate, undergo apoptosis or senescence [27-40]. Phosphatidylinositol (PI) (3,4)P_2_ and PI(3,4,5)P_3_ produced by class 1A PI3Ks recruit phosphoinositide dependent kinase-1 (PDK1) as well as Akt isoforms to the plasma membrane by interacting with their pleckstrin homology (PH) domains [41-44]. Co-localization of PDK1 with Akts at the plasma membrane causes PDK1 to phosphorylate Akts at a threonine residue (T308) and a serine residue (S473). The kinase which phosphorylates S473 is believed to be mTORC2, which actually is the rapamycin-insensitive complex which lies downstream of Akt [44]. Activation of PDK1 and Akt by class 1A PI3Ks is negatively regulated by phosphatase and tensin homologue deleted on chromosome ten (PTEN) [7, 9, 44-48]. PTEN removes phosphate groups from PI(3,4)P_2_ and PI(3,4,5)P_3_ added by PI3K as well as from tyrosine phosphorylated proteins including focal adhesion kinase (FAK) and Shc [7,11,44]. PTEN is a critical tumor suppressor gene and another key lynch-pin in regulation of this pathway. PTEN is frequently inactivated by various genetic mechanisms which results in Akt activation.

Diverse mechanisms regulate PTEN expression [10,47-50]. These range from gene deletion, alterations in mRNA splicing, subcellular localization or epigenetic mechanisms which prevent PTEN transcription. There are many microRNAs (miRNAs) which target the PTEN gene to inhibit its expression [10,47,51]. Furthermore there is a pseudo PTEN gene which serves as a decoy to bind and neutralize some of these miRNAs [10,47]. Mutations have been reported to occur at PTEN in breast cancer at varying frequencies (5-21%). While PTEN is deleted in certain cancers, loss of heterozygosity (LOH) is probably a more common genetic event (30%) leading to changes in PTEN expression [47,52]. PTEN promoter methylation leads to low PTEN expression [47]. In one study, 26% of primary breast cancers had low PTEN levels which correlated with lymph node metastases and poor prognoses [53]. PTEN has both plasma membrane and nuclear localized activities. Disruption of PTEN activity by various mechanisms frequently results in Akt activation. Activation of Akt could have vast effects on different processes affecting breast cancer drug resistance and sensitivity to targeted therapy.

A consequence of impaired PTEN expression is elevated activation of Akt. Elevated Akt activity can have many effects on cell growth, such as; the phosphorylation and activation/inactivation of transcription factors which control pivotal gene expression, the inactivation of anti-apoptotic molecules by their phosphorylation and subsequent protesomal degradation, or by the regulation of translation of key “weak” mRNAs involved in growth. One downstream molecule of mTOR is ribosomal S6 kinase (p70S6K). This kinase regulates the efficiency of translation of certain mRNAs and also functions in a negative feedback loop to control Akt activity [10,44,54-55]. The roles of Akt and p70S6K and other molecules are essential for the formation of the eIF4F translation complex. The eIF4F complex is necessary for the translation of mRNAs containing long 5’UTRs which are highly-structured and have an elevated G+C content. These mRNAs are considered “weak” mRNAs. These “weak” mRNAs often encode genes involved in oncogenesis and survival such as c-Myc, Mcl-1, cyclin-D, VEGF and survivin. Furthermore p70S6K has important roles in autophagy and cellular senescence [56].

Akt, mTOR and p70S6K activation have been associated with a more severe prognosis in breast and other cancers [53,57-64]. Targeting the PI3K/PTEN/Akt/mTOR pathway may prove effective in various cancer therapies [12,54,65-66]. High levels of activated Akt expression have been associated with both chemo- and hormonal resistance in breast cancer [58-59,67]. Indeed some studies have evaluated the effectiveness of targeting mTOR in PTEN-negative cells [60]. Cells which express high levels of activated Akt may be more sensitive to mTOR inhibitors and inhibition of mTOR activity by rapamycin may restore their sensitivity to chemo- and hormonal based therapies [9,60]. A distinct advantage to targeting mTOR with rapamycin is that rapamycin has been used many years to treat organ transplant patients (especially kidney transplant). Rapamycin is now being examined in treatment of certain cancers and in the prevention of aging and other diseases including AIDS [11-12,68].

Previously it was determined that mutated forms of Akt and PTEN can induce chemotherapeutic- and hormonal-based drug resistance in breast cancer [9,58,67]. PTEN mutation which eliminate lipid phosphatase activity will result in activated Akt expression which will lead to drug resistance and sensitivity to the mTOR inhibitor rapamycin [9].

In the following studies, the effects enforced expression of activated Akt-1 on drug resistance and sensitivity to targeted therapy. Inhibitors targeting mTOR suppressed drug resistance demonstrating the importance of mTOR in chemosensitivity. These experiments were performed to understand the effect of common chemotherapeutic drugs on anti-apoptotic signaling pathways as well as how these signaling pathways can change in drug resistant cells.

## RESULTS

### Effects of Constitutively-Activated Akt-1 Expression on the Sensitivity of MCF-7 Cells to Chemotherapeutic Drugs

**Figure 1:**
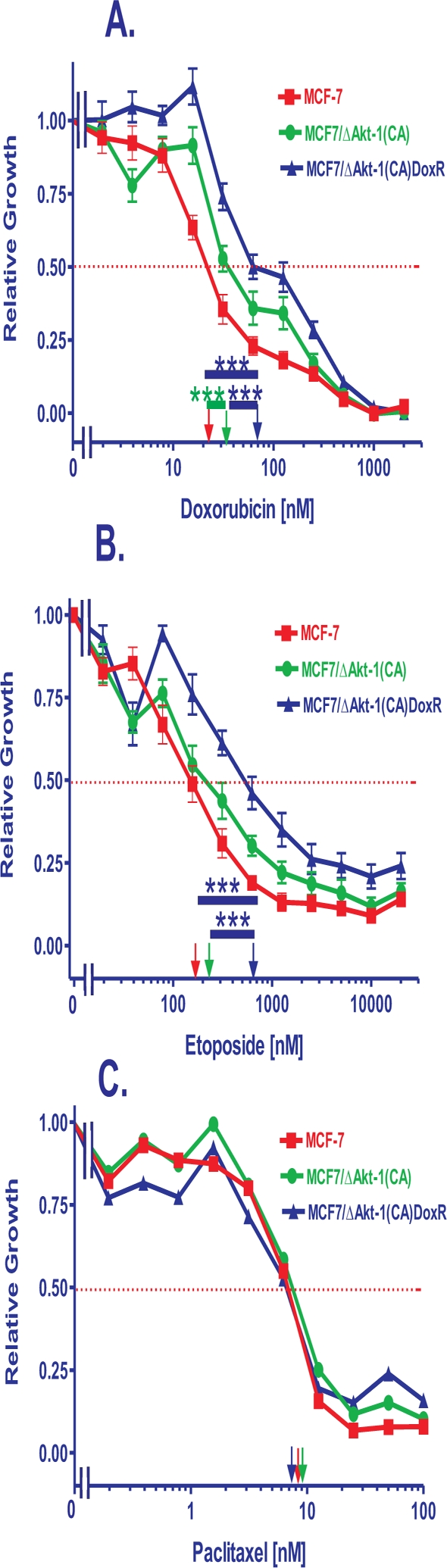
Effects of Akt-1 Expression on Chemotherapeutic Drug Sensitivity The effects of Akt-1(CA) expression on sensitivity of MCF-7 cells to: **Panel A.** doxorubicin, **Panel B.** etoposide and **Panel C.** paclitaxel were examined by MTT analysis after incubation of the cells in the indicated concentrations of the drugs. MCF7/ΔAkt-1(CA) were isolated after infection of MCF-7 cells with a retrovirus encoding ΔAkt-1(CA) and selection in G418 (geneticin) for 4 weeks. MCF7/ΔAkt-1(CA)DoxR cells were isolated after selection of MCF7/ΔAkt-1(CA) cells for 4 weeks in medium containing 25 nM doxorubicin. The dotted arrow represents where 50% inhibition of growth intercepts with the X axis and is used to estimate the IC_50_. The statistical significance was determined by the unpaired *t* test. Comparisons determined to be significant are indicated with (***). Panel A. The *P* value between doxorubicin treatment of MCF-7 and MCF7/ΔAkt-1(CA) cells was 0.0025. The *P* value between doxorubicin treatment of MCF-7 and MCF7/ΔAkt-1(CA)DoxR cells was <0.0001. The *P* value between doxorubicin treatment of MCF7/ΔAkt-1(CA) and MCF7/ΔAkt-1(CA)DoxR cells was 0.0003. Panel B. The *P* value between etoposide treatment of MCF-7 and MCF7/ΔAkt-1(CA)DoxR cells was 0.0003. The *P* value between etoposide treatment of MCF7/ΔAkt-1(CA) and MCF7/ΔAkt-1(CA)DoxR cells was 0.001.

A means to examine the effects of deregulated Akt-1 expression on the sensitivity of MCF-7 cells to chemotherapeutic- and hormonal-based drugs is to infect MCF-7 cells with a retrovirus encoding a constitutively-active Akt gene such as ΔAkt-1(CA) [69]. Pools of ΔAkt-1(CA)-infected MCF-7 cells were recovered after selection in medium containing G418 as that is the drug that the *neoR* gene present in the retroviral vector encodes resistance to. The ΔAkt-1(CA)-infected cells are named MCF7/ΔAkt-1(CA). The sensitivities of these cells to chemotherapeutic drugs were determined by MTT analysis after incubation for 4 days in the indicated culture conditions. Figure 1 illustrates the sensitivities of the MCF-7 and MCF7/ΔAkt-1(CA) cells to doxorubicin, etopside and paclitaxel. As an additional control, similar experiments were performed with MCF-7 cells transfected with the empty *neo*r plasmid, pEGFP (MCF7/EGFP). These cells represent a control for cells transfected with just a *neoR* gene. MCF7/EGFP cells were similar in drug sensitivity as MCF-7 cells.

Initially, the ΔAkt-1(CA)-infected cells were about 2- and 1.5-fold more resistant to doxorubicin (Figure 1, Panel A) and etoposide (Figure 1, Panel B) respectively than the parental MCF-7 (Table 1). In contrast the MCF7/ΔAkt-1(CA) cells did not show any difference in sensitivity to paclitaxel than the parental MCF-7 cell line from which they were derived (Figure 1, Panel C).

**Table 1:** Effects of Activated Akt-1 Expression on Chemotherapeutic and Hormonal Drugs and Signal Transduction Inhibitors IC50s.^1^

Cell Line	Drug Treatment	IC502 or Change in IC503
MCF-7	Doxorubicin	20 nM2
MCF-7/ΔAkt-1(CA)	Doxorubicin	2 X↑^3^
MCF-7/ΔAkt-1(CA)DoxR	Doxorubicin	6.7 X↑^3^
MCF-7	Etoposide	140 nM^2^
MCF-7/ΔAkt-1(CA)	Etoposide	1.5 X↑^3^
MCF-7/ΔAkt-1(CA)DoxR	Etoposide	3.1 X↑^3^
MCF-7	Paclitaxel	10 nM^2^
MCF-7/ΔAkt-1(CA)	Paclitaxel	-^3^
MCF-7/ΔAkt-1(CA)DoxR	Paclitaxel	-^3^
MCF-7	4HT	7 nM^2^
MCF-7/ΔAkt-1(CA)	4HT	4.3 X↑^3^
MCF-7/ΔAkt-1(CA)DoxR	4HT	10 X↑^3^
MCF-7	Rapamycin	7 nM^2^
MCF-7/ΔAkt-1(CA)	Rapamycin	2.5 X↓^3^
MCF-7/ΔAkt-1(CA)DoxR	Rapamycin	7 X↓^3^

^1^Determined by MTT analysis as presented in Figures 1 and 2.

^2^IC_50_ for MCF-7 cells.

^3^Comparisons to value obtained in MCF-7 cells. - = baseline with MCF-7 cells or no difference compared to MCF-7 cells. X↑ = fold increase in IC_50_. X↓ = fold decrease in IC_50_.

We also isolated ΔAkt-1(CA) cells with increased resistance to chemotherapeutic drugs by culturing the cells for 4 weeks in medium containing 25 nM doxorubicin. These cells were named MCF7/ΔAkt-1(CA)DoxR and they were 5-fold more frequently recovered from cells infected with the ΔAkt-1(CA)-containing virus than from control cell lines. The MCF7/ΔAkt-1(CA)DoxR cells were 6.7- and 3.1-fold more resistant to doxorubicin and etoposide respectively than control MCF-7 cells (Figure 1, Panels A & B). In contrast, no difference in sensitivity to paclitaxel was observed (Figure 1, Panel C). The IC_50_s for doxorubicin ranged from approximately 10 to 120 nM in the doxorubicin sensitive MCF-7 and resistant MCF7/ΔAkt-1(CA)DoxR cells respectively. The IC_50_s for etoposide ranged from 150 to 600 nM in the doxorubicin sensitive MCF-7 and resistant MCF7/ΔAkt-1(CA)DoxR cells the respectively. In contrast, the IC_50_s for paclitaxel were approximately 10 nM in the doxorubicin sensitive MCF-7 and resistant MCF7/ΔAkt-1(CA)DoxR cells.

### Effects of Constitutively-Activated Akt-1 Expression of the Sensitivity of MCF-7 Cells to the Hormone-Based Drug Tamoxifen

**Figure 2:**
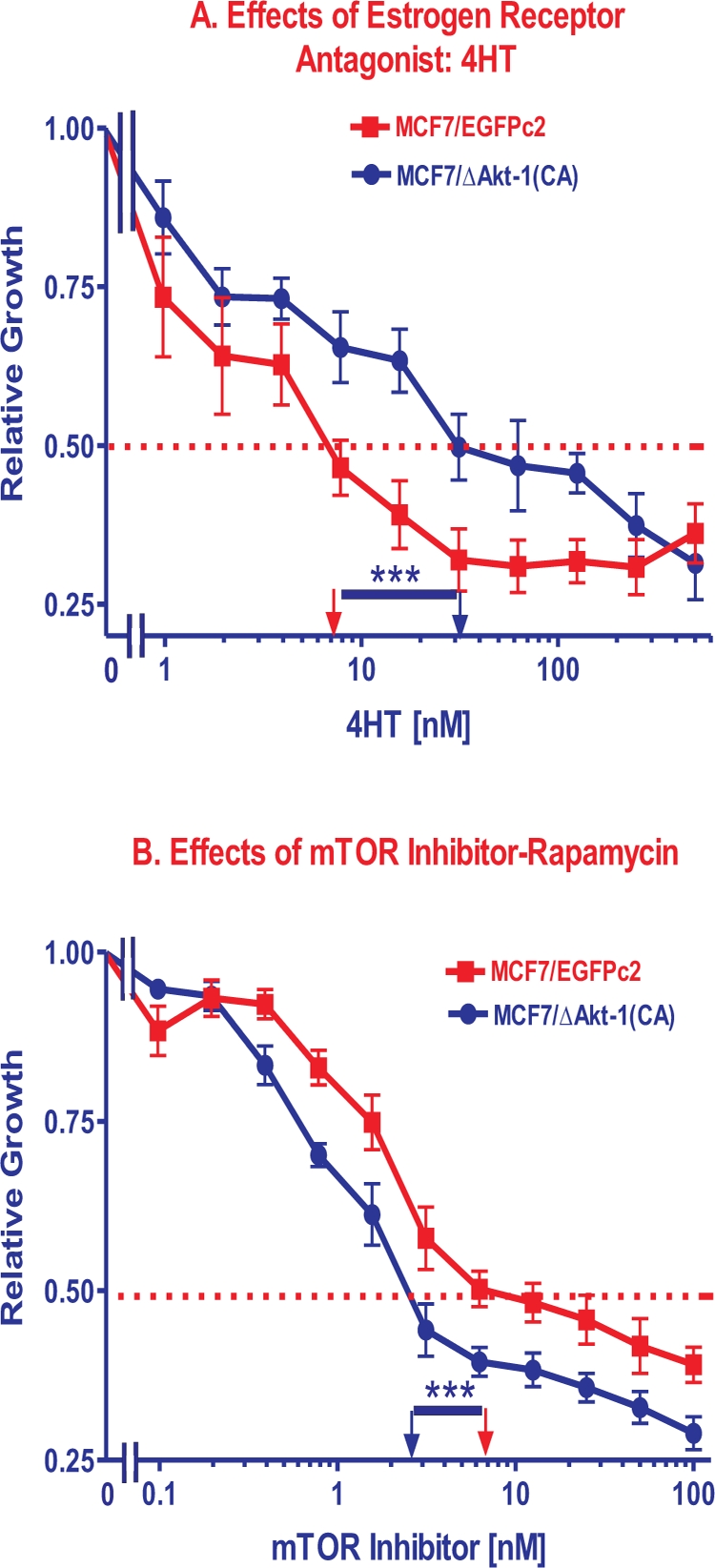
Effects of Akt Expression on Sensitivity to Tamoxifen and Rapamycin The effects of ΔAkt-1(CA) expression on sensitivity of MCF-7 cells to: **Panel A.** tamoxifen (4HT) and **Panel B.** rapamycin was examined by MTT analysis after incubation of the cells in the indicated concentrations of the drugs. The dotted arrow represents where 50% inhibition of growth intercepts with the X axis and is used to estimate the IC_50_. The statistical significance was determined by the unpaired *t* test. Comparisons determined to be significant are indicated with (***). Panel A. The *P* value between 4HT treatment of MCF7/EGFPc2 and MCF7/ΔAkt-1(CA) cells was 0.005. The *P* value between rapamycin treatment of MCF7/EGFPc2 and MCF7/ΔAkt-1(CA) cells was 0.019.

The effects of the hormonal drug 4-hydroxytamoxifen (4HT) were examined on the estrogen receptor positive (ER+) MCF-7 and MCF7/ΔAkt-1(CA) cells by MTT analysis (Figure 2, Panel A). The IC_50_s for 4HT in the ER+ MCF-7 and Akt-transduced cells ranged from 7 to 200 nM (Table 1). The MCF7/ΔAkt-1(CA) cells were approximately 4.3-fold more resistant to the effects of 4HT than MCF-7 cells. The MCF7/ΔAkt-1(CA)DoxR cells were approximately 10-fold more resistant the effects of 4HT than MCF-7 cells (Table 1). Thus constitutive Akt-1 expression elevated the concentration of 4HT required to achieve the IC_50_.

### Effects of Constitutively-Activated Akt-1 Expression of the Sensitivity of MCF-7 Cells to the mTOR Inhibitor Rapamycin

Constitutive expression of activated Akt-1 conferred increased sensitivity to the mTOR inhibitor rapamycin as the IC_50_s for rapamycin were 2.8- and 7-fold lower in MCF-7/ΔAkt-1(CA) and MCF-7/ΔAkt-1(CA)DoxR, respectively, than in MCF-7 cells (Figure 2, Panel B and Table 1). The IC_50_s for rapamycin ranged from approximately 1 to 10 nM. MCF-7 cells are inherently sensitive to rapamycin, perhaps due to a mutation at *PIK3CA* in this cell line [23]. The doxorubicin-selected MCF-7/ΔAkt-1(CA)DoxR cells were 2.5-fold more sensitive to rapamycin than the MCF-7/ΔAkt-1(CA) cells. Thus introduction of activated Akt-1 into MCF-7 cells conferred increased sensitivity to rapamycin and drug resistant MCF-7/ΔAkt-1(CA)DoxR cells were even more sensitive to rapamycin.

### Enhancement of Doxorubicin Induced-Inhibition of Proliferation by Addition of Tamoxifen

**Figure 3:**
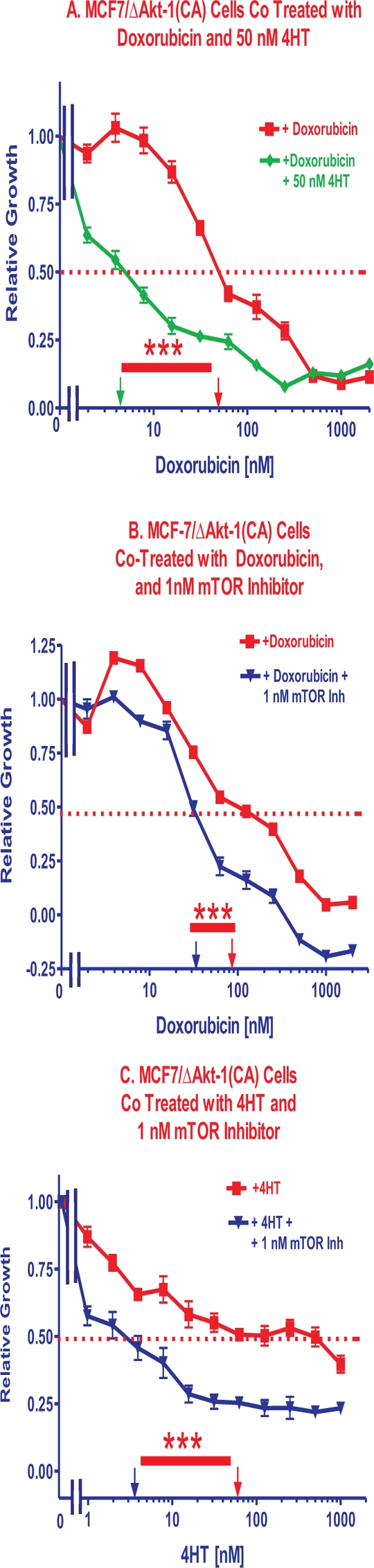
Effects of Co-Treatment with Doxorubicin, 4HT and a mTOR Inhibitor. Panel A The effects of adding a constant dose of 50 nM 4HT on the doxorubicin IC_50_ were examined in MCF7/ΔAkt-1(CA) cells. **Panel B.** The effects of adding a constant dose of 1 nM rapamycin on the doxorubicin IC_50_ were examined in MCF7/ΔAkt-1(CA) cells. **Panel C.** The effects of adding a constant dose of 1 nM rapamycin on the 4HT IC_50_ were examined in MCF7/ΔAkt-1(CA) cells. The statistical significance was determined by the unpaired *t* test. Comparisons determined to be significant are indicated with (***). Panel A. The *P* value between doxorubicin vs. doxorubicin and 50 nM 4HT treatment of MCF7/ΔAkt-1(CA) cells was 0.0001. Panel B. The *P* value between doxorubicin vs. doxorubicin and 1 nM rapamycin treatment of MCF7/ΔAkt-1(CA) cells was 0.0008. Panel C. The *P* value between 4HT vs. 4HT and 1 nM rapamycin treatment of MCF7/ΔAkt-1(CA) cells was 0.0001.

The abilities to improve chemotherapy by potentially lowering dose-associated deleterious side effects were examined. Namely, we determined the ability of hormonal-based therapy to lower the doxorubicin IC_50_. In this particular experiment presented in Figure 3, Panel A, we added a constant dose of 50 nM 4HT and different doses of doxorubicin to examine the effects on MCF7/ΔAkt-1(CA) cells. It should be pointed out that 4HT by itself, like rapamycin, did not totally eliminate viability of the cells after 4 days of incubation, as approximately 25% relative growth was still observed (Figure 2, Panels A & B). In contrast, high concentrations of doxorubicin (>500 nM) almost totally eliminated the relative growth of the cells at sufficient concentrations (Figure 1). As shown in Figure 3, Panel A, addition of 50 nM 4HT reduced the doxorubicin IC_50_ approximately 10-fold. Moreover, when the doses of doxorubicin required for essentially total cell death (>95% inhibition) were examined, a 2-fold lower dose of doxorubicin was required (250 vs 500 nM doxorubicin) when 4HT was added than in its absence.

Next we examined the effects of a constant, sub-optimal dose of 5 nM 4HT on the doxorubicin IC_50_s in MCF-7, MCF-7/ΔAkt-1(CA), and MCF-7/ΔAkt-1(CA)DoxR cells. 7.5-, 10.7- and 8.9-fold decreases in the IC_50_ for doxorubicin were observed (Table 2). These results indicated that suppressing the ER could promote the cytotoxic effects of doxorubicin at lower concentrations. Moreover, even though the MCF-7/ΔAkt-1(CA) cells required more 4HT to reach the IC_50_ than MCF-7 cells, they remained sensitive to 4HT.

**Table 2:** Effects of Combination of Chemotherapeutic, Hormone Based and Signal Transduction Inhibitors IC50s.^1^

Cell Line	Drug Treatment	Change in IC502
MCF-7	Doxorubicin + 5 nM 4HT	7.5 X↓^2^
MCF-7/ΔAkt-1(CA)	Doxorubicin + 5 nM 4HT	10.7 X↓^2^
MCF-7/ΔAkt-1(CA)DoxR	Doxorubicin + 5 nM 4HT	8.9 X↓^2^
MCF-7	Doxorubicin + 1 nM Rapamycin	-^2^
MCF-7/ΔAkt-1(CA)	Doxorubicin + 1 nM Rapamycin	2.5 X↓^2^
MCF-7/ΔAkt-1(CA)DoxR	Doxorubicin + 1 nM Rapamycin	60 X↓^2^
MCF-7	Etoposide + 1 nM Rapamycin	-^2^
MCF-7/ΔAkt-1(CA)	Etoposide + 1 nM Rapamycin	1.3 X↓^2^
MCF-7/ΔAkt-1(CA)DoxR	Etoposide + 1 nM Rapamycin	2.5 X↓^2^
MCF-7	4HT + 1 nM Rapamycin	5.8 X↓^2^
MCF-7/ΔAkt-1(CA)	4HT + 1 nM Rapamycin	18.7 X↓^2^
MCF-7/ΔAkt-1(CA)DoxR	4HT + 1 nM Rapamycin	60 X↓^2^

^1^ Determined by MTT analysis as presented in Figures 3, 4 & 5.

^2^ Comparison with identical cell line treated the same day with just single chemotherapeutic- or hormone based drug, averages of 3 experiments (See Figures 4 and 5 for examples). - = no difference compared to same cell line. X↑ = fold increase in IC_50_. X↓ = fold decrease in IC_50_.

### Enhancement of Doxorubicin and 4HT Induced Inhibition of Proliferation by the mTOR Inhibitor Rapamycin

The abilities to improve chemo- and hormonal-based therapy by potentially lowering dose-associated deleterious side effects were examined. Namely, we examined the ability of the mTOR inhibitor rapamycin to lower the doxorubicin and 4HT IC_50_s. In these experiments, we used a constant doses of 1 nM mTOR inhibitor and different doses of either 4HT or doxorubicin to examine the effects on MCF7/Akt-1(CA) cells (Figure 3, Panels B and C). Co-addition of constant doses of the mTOR inhibitor lowered the doses of doxorubicin and 4HT required to obtain the doxorubicin and 4HT IC_50_s by 2.6- and 14-fold respectively. However, even with the mTOR inhibitor, 4HT did not totally eliminate the relative growth of MCF7/ΔAkt-1(CA) cells (Panel C) while doxorubicin did (Panel B).

Combinatorial effects on 4HT IC_50_s were observed with rapamycin in MCF-7, MCF7/ΔAkt-1(CA) and MCF7/ΔAkt-1(CA)DoxR cells (Table 2). The highest level of rapamycin-mediated suppression of the 4HT IC_50_s was observed with the MCF7/ΔAkt-1(CA)DoxR cells although rapamycin-mediated suppression of the 4HT IC_50_s was also observed in doxorubicin sensitive MCF-7 cells. Thus rapamycin is able to synergize with 4HT in these ER^+^ ce^l^ls (Table 2).

### Effects of mTOR Inhibitors on Sensitivity of MCF-7 Cells to Doxorubicin

**Figure 4:**
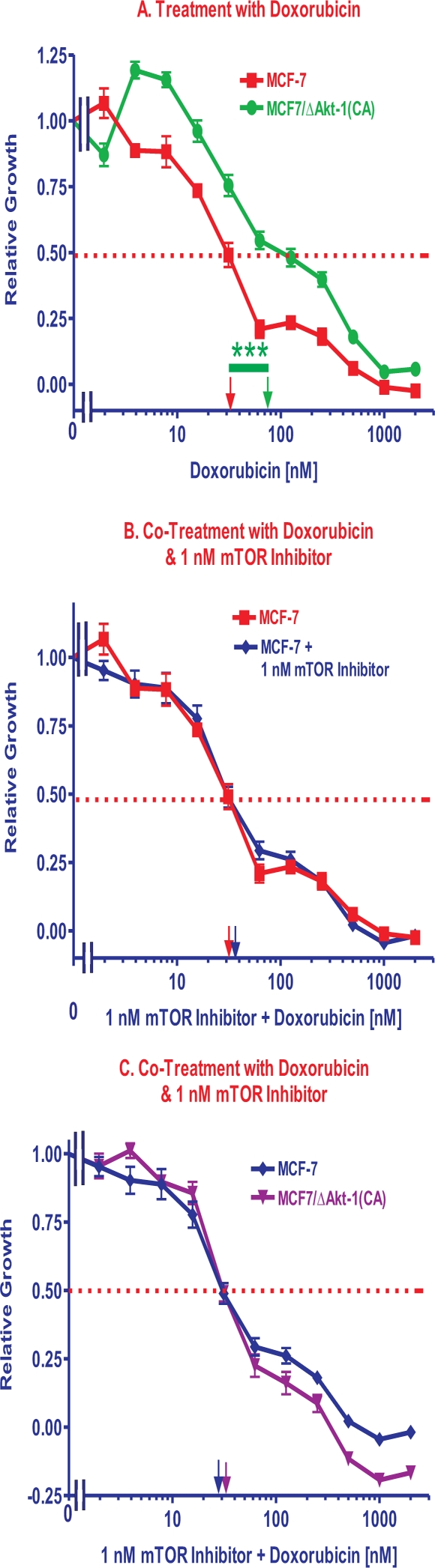
Effects of Co-Treatment with Doxorubicin and a mTOR Inhibitor. Panel A The sensitivities of MCF-7 and MCF7/ΔAkt-1(CA) cells to different doses of doxorubicin in presented. **Panel B.** The sensitivity of MCF-7 cells to different doses of doxorubicin is compared with the effects of doxorubicin and a suboptimal dose of 1 nM rapamycin. **Panel C.** The sensitivities of MCF-7 and MCF7/ΔAkt-1(CA) cells to different doses of doxorubicin and a suboptimal dose of 1 nM rapamycin in presented. The statistical significance was determined by the unpaired *t* test. Comparisons determined to be significant are indicated with (***). Panel A. The *P* value between doxorubicin treatment of MCF-7 and MCF7/ΔAkt-1(CA) cells was 0.0005.

MCF-7 cells were more sensitive to doxorubicin than MCF7/ΔAkt-1(CA) cells (Figure 4, Panel A). However, low doses of rapamycin had greater effects on the doxorubicin IC_50_s in MCF7/ΔAkt-1(CA) than in MCF-7 cells. Essentially a constant dose of 1 nM rapamycin did not reduce the IC_50_ for doxorubicin in MCF-7 cells (Figure 4, Panel B), while it did reduce the IC_50_ for doxorubicin in MCF7/ΔAkt-1(CA) cells to approximately the same IC_50_ level observed in MCF-7 cells (Panel C).

### Effects of mTOR Inhibitors on Sensitivity of MCF-7 and MCF7/ΔAkt-1(CA) Cells to Etoposide and 4HT

**Figure 5:**
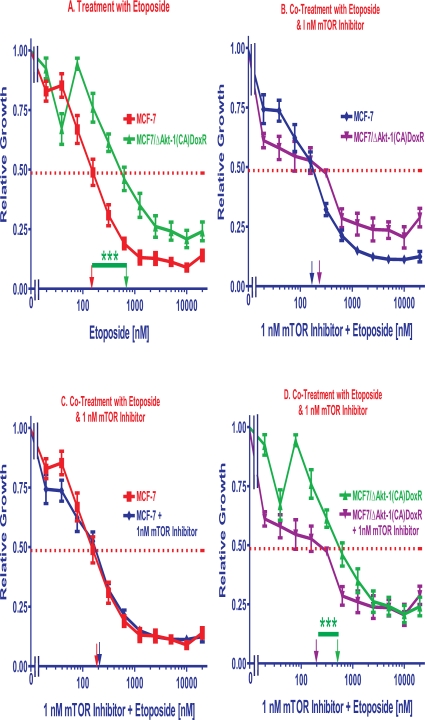
Effects of Co-Treatment with Etoposide and Rapamycin on Etoposide IC_50_s The effects of adding a sub-optimal constant dose of 1 nM rapamycin on the etoposide IC_50_s were examined in MCF-7 and MCF7/ΔAkt-1(CA)DoxR cells. **Panel A**. The sensitivity of MCF-7 and MCF7/ΔAkt-1(CA)DoxR cells to different doses of etoposide in presented. **Panel B.** The sensitivities of MCF-7 and MCF7/ΔAkt-1(CA)DoxR cells to different doses of etoposide and a suboptimal dose of 1 nM rapamycin in presented. **Panel C.** The sensitivity of MCF-7 cells to different doses of etoposide is compared with the effects of etoposide and a suboptimal dose of 1 nM rapamycin. **Panel D.** the sensitivity of MCF7/ΔAkt-1(CA)DoxR cells to different doses of etoposide is compared with the effects of etoposide and a suboptimal dose of 1 nM rapamycin. Comparisons determined to be significant are indicated with (***). Panel A. The *P* value between etoposide treatment of MCF-7 and MCF7/ΔAkt-1(CA)DoxR cells was 0.0003. Panel D. The *P* value between etoposide and etoposide and 1nM rapamycin treatment of MCF7/ΔAkt-1(CA)DoxR cells was 0.0005.

MCF-7 cells were more sensitive to etoposide than MCF-7/ΔAkt-1(CA)DoxR cells (Figure 5, Panel A). Rapamycin was able to lower the concentration of etoposide required to reach the IC_50_ in the MCF-7/ΔAkt-1(CA) and MCF-7/ΔAkt-1(CA)DoxR cells (Figure 5 and Table 2). Usually a higher degree of interaction was observed in the MCF-7/ΔAkt-1(CA)DoxR cells. The results of addition of 1 nM rapamycin on the etoposide IC_50_s in MCF-7 and MCF-7/ΔAkt-1(CA)DoxR cells are presented in Panel B. Rapamycin suppressed the etoposide IC_50_s the most in the MCF-7/ΔAkt-1(CA)DoxR cells, with approximately a 2-fold reduction as compared to MCF-7 cells (Table 2). 1 nM rapamycin did not suppress the IC_50_s of etoposide in MCF-7 cells (Panel C), but did suppress the IC_50_ for etoposide in MCF-7/ΔAkt-1(CA)DoxR cells approximately 2.5 fold (Panel D).

Rapamycin also suppressed the 4HT IC_50_s in the doxorubicin-sensitive and resistant- cells (Table 2). The highest level of rapamycin-mediated suppression of the 4HT IC_50_s was observed with the MCF-7/ΔAkt-1(CA)DoxR cells although rapamycin-mediated suppression of the 4HT IC_50_s was also observed in doxorubicin-sensitive MCF-7 cells.

## DISCUSSION

In our studies, we examined the effects of three chemotherapeutic drugs (doxorubicin, etoposide and paclitaxel), one hormonal drug (tamoxifen, 4HT) and the signal transduction inhibitor rapamycin on MCF-7 and derivative cell lines which varied in their levels of activated Akt-1 expression. An advantage of our study is that all the cells had the same genetic background as they all were MCF-7 breast cancer cells, however they differed in the levels of activated Akt-1 expression due to introduction of an activated Akt-1 gene.

We have previously shown that introduction of dominant negative (DN) forms of PTEN into MCF-7 cells conferred resistance to doxorubicin and increased sensitivity to rapamycin [9]. Furthermore, rapamycin could synergize with doxorubicin to lower its IC_50_ [9]. In the MCF-7 cells transfected cells with the DN PTEN genes, increased levels of activated Akt were detected. However, these drug-resistant PTEN(DN) transfected cells were hypersensitive to rapamycin. These results have clinical significance as the PI3K/PTEN/Akt/mTOR pathway is often activated in breast cancer by mutations at *PIK3CA* or multiple genetic mechanisms leading to dysregulation of PTEN. Furthermore, drug resistance frequently develops in breast cancer after chemo- or hormonal-based therapies. Doxorubicin (a.k.a Adriamycin) is frequently used to treat breast cancer patients. In the studies present in this report, increased expression of activated Akt-1 resulted in the resistance of MCF-7 breast cancer cells to both chemotherapeutic drugs (doxorubicin and etoposide) as well as hormonal based drugs (4HT) and these drug resistant activated Akt-1 overexpressing cells were also hypersensitive to rapamycin, which reduced the concentrations of doxorubicin, etoposide and 4HT required to reach the IC_50_s in these cells.

The roles of Akt and downstream mTOR are critical. Hyperactivation of mTOR will induce cellular senescence which can be inhibited by mTOR inhibitors. mTOR appears to be an essential switch which stimulates p53 to promote cellular quiescence vs. senescence. [70-75].

Doxorubicin, etoposide and paclitaxel all have been used to treat breast cancer. However, doxorubicin and paclitaxel are more commonly employed in breast cancer therapy today than etoposide [76]. Tamoxifen is a common hormonal-based drug used to treat ER+ breast cancer. After chemotherapeutic drug and hormonal based drug treatment of breast cancer patients, resistance will develop [10,77]. Thus it is essential to develop new more effective and alternative methods to treat breast cancer.

Doxorubicin, etoposide and paclitaxel can all be transported by the same drug transporter (Mdr-1, a.k.a., Pgp, and *ABCB1*) [78]. Our results suggest that Mdr1 is not likely to be involved in the drug resistance of these cells but our studies do not exclude many other drug transporters. Both doxorubicin and etoposide target events involved in DNA replication, whereas paclitaxel targets microtubules. Therefore it is not surprising that cells selected for resistance to doxorubicin are also resistant to etoposide, but not paclitaxel. Our studies suggest that attention should be given during treatment of breast cancer patients who have become resistant to a particular chemotherapeutic drug, as these patients may be cross-resistant to other chemotherapeutic drugs which target similar events.

Interestingly, suboptimal doses of 4HT would interact with doxorubicin to lower the doxorubicin IC_50_ in MCF-7, MCF-7/ΔAkt-1(CA), and MCF-7/ΔAkt-1(CA)DoxR approximately 8- to 10-fold. This demonstrates the important effects of 4HT on ER cells. Even though cells that express activated Akt require more 4HT to reach the IC_50_ than cells which do not express activated Akt, they will still respond to 4HT when they are also treated with doxorubicin. Thus resistance to doxorubicin does not prevent a response to 4HT.

The mechanisms by which doxorubicin and rapamycin synergize to inhibit the growth of breast cancer cells are under further investigation. Doxorubicin can induce reactive oxygen species (ROS) which have many effects on cell growth and the prevention of apoptosis. A consequence of doxorubicin treatment may be the induction of the Raf/MEK/ERK pathway which may have some anti-apoptotic effects [79].

The ability of a 1 nM constant addition of the mTOR inhibitor rapamycin to interact with 4HT and lower the IC_50_ for 4HT was observed in MCF-7 and MCF-7/ΔAkt-1(CA) cells. In contrast, 1 nM rapamycin did not increase the sensitivity of MCF-7 cells to either doxorubicin or etoposide. Higher doses of the mTOR inhibitor were not examined in these studies as 1 nM rapamycin was close to the IC_50_s previously observed in the _c_ell lines. 1 nM rapamycin was sufficient to increase the sensitivity of MCF-7/ΔAkt-1(CA) cells and not MCF-7 cells to doxorubicin and etoposide.

These results are relevant to potential cancer therapies as Akt is frequently activated by upstream *PIK3CA* or *PTEN* mutations or gene silencing. *PTEN* can be mutated or silenced by various mechanisms in human cancer. Mutations occur which either delete the *PTEN* gene or alter its activity. Sometimes these mutations actually make the cells sensitive to Akt and mTOR inhibitors as the growth of the cells becomes dependent upon elevated Akt levels and downstream mTOR and p70S6K activities [60].

## MATERIALS AND METHODS

### Cell Culture

MCF-7 cells were obtained from the American Type Culture Collection (ATCC) (Manassas, VA). Cell culture medium for MCF-7 cells consisted of Roswell Park Memorial Institute-1640 (RPMI 1640) medium (Invitrogen. Carlsbad CA) supplemented with 10% (v/v) heat inactivated fetal bovine serum (FBS) as described [5].

### Akt Plasmids

MCF-7 cells were infected with a retrovirus encoding the ΔAkt-1(CA) gene [69,80]. Stably ΔAkt-1(CA)- infected or EGFP-transfected MCF-7 cells were isolated after growth for 4 weeks in selective drug (2 mg/ml G418, Sigma-Aldrich, St. Louis, MO) as described [9,80].

### Analysis of Sensitivity to Drugs and Signal Transduction Inhibitors.

Cells were seeded in 96-well cell culture plates (BD Biosciences) at a density of 5,000 cells/well in 100 μl/well of phenol red free RPMI-1640 containing 5% charcoal stripped (CS) FBS as described [5]. Cells were incubated for 1 day to permit cells to adhere to the bottom of each well. Cells were subsequently treated with serial 2-fold dilutions of the chemotherapeutic drugs (doxorubicin, etoposide or paclitaxel), the hormonal drug (4HT), signal transduction inhibitor, rapamycin, (all purchased from Sigma-Aldrich) or a constant suboptimal concentration of the hormonal drug or signal transduction inhibitor. Cells were incubated for an additional 4 days at 37 °C until extent of 3-(4,5-dimethylthiazol-2-yl)-2,5-diphenyl-2*H*-tetrazolium bromide (MTT, Sigma-Aldrich) reduction in each well was quantified at 530 nm [5].
